# Checkpoint Inhibition May Trigger the Rare Variant of Anti-LAD-1 IgG-Positive, Anti-BP180 NC16A IgG-Negative Bullous Pemphigoid

**DOI:** 10.3389/fimmu.2019.01934

**Published:** 2019-08-14

**Authors:** Christian D. Sadik, Ewan A. Langan, Victoria Grätz, Detlef Zillikens, Patrick Terheyden

**Affiliations:** Department of Dermatology, Allergy, and Venereology, University of Lübeck, Lübeck, Germany

**Keywords:** checkpoint inhibitors, bullous pemphigoid, LAD-1, skin inflammation, melanoma, nivolumab

## Abstract

Bullous pemphigoid (BP) is an autoimmune blistering skin disease characterized by an autoimmune response to type XVII collagen (BP180). The generation of anti-BP180-NC16A IgG autoantibodies is considered to be central to the pathogenesis of BP, in part due to the close correlation between serum concentration and disease activity. However, ~60% of BP patients also generate IgG autoantibodies against LAD-1, the soluble 120 kDa ectodomain of BP180. Whilst the pathogenic significance of anti-LAD-1 IgG remains unclear, it may be sufficient to precipitate the development of BP, even in the absence of anti-BP180-NC16A IgG, based on several case reports in Japanese patients. There is increasing recognition that immune-checkpoint inhibitors may trigger and/or exacerbate BP as an immune-related adverse event (irAE). Until now, all of these cases have been associated with the induction of anti-BP180-NC16A IgG. Here, we report the case of a female Caucasian patient who developed BP during treatment with the programmed cell death protein 1 (PD-1) inhibitor nivolumab. Intriguingly, the patient exclusively generated anti-LAD-1 IgG, suggesting that anti-LAD-1 IgG was responsible for the development of her autoimmune blistering dermatosis. This is the first such case documented in a non-Japanese patient, thus, lending further support to the pathogenic relevance of anti-LAD-1 IgG in BP.

## Introduction

Nivolumab, an immune checkpoint inhibitor, is an IgG_4_ antibody which antagonizes the programmed cell death protein 1 (PD-1) receptor (1) and was licensed by the FDA in 2014 for the treatment of metastatic melanoma (1). It has subsequently been approved for the treatment of a wide variety of malignant tumors, including small and non-small cell lung cancer, head and neck cancer, urothelial tract cancer, Hodgkin's lymphoma, hepatocellular, and renal cell carcinoma (2). As with other checkpoint inhibitors, the major side-effects are termed “immune-related adverse events” (irAEs) and present with organ-specific tissue inflammation (1). In fact, up to 42% of patients receiving nivolumab develop cutaneous irAEs (1), ranging from itch to maculopapular, lichenoid, eczematous, granulomatous, or erythema multiforme-like skin changes (3, 4).

There is now increasing evidence that checkpoint inhibition can also result, in rare cases, in the development of blistering skin diseases; clinically and immunopathologically indistinguishable from bullous pemphigoid (BP) (5–14) and associated with the development of tense blisters and erosions on erythematous skin. Histologically, these lesions are characterized by subepidermal clefts and immunofluorescence reveals linear deposition of IgG and C3 at the dermal-epidermal junction (DEJ). In most cases, circulating IgG autoantibodies, directed to the NC16A domain of the hemidesmosomal protein BP180 (type XVII collagen), are detectable in the serum. The presence of these antibodies supports the clinical observation that checkpoint inhibitors can trigger BP.

The immunodominant autoantigen in spontaneous BP is BP180-NC16A with anti-BP180-NC16A IgG detectable in the serum of ~90% of patients with BP (15–17). In addition, 60–70% of patients with BP also produce IgG autoantibodies against the intracellular hemidesmosomal plaque protein BP230 (15). Approximately 60% of these patients also generate antibodies targeting the 120 kDa soluble ectodomain (LAD-1) of BP180 (18, 19), but the significance of these antibodies in the aetiopathogenesis of BP remains uncertain (20).

Interestingly, evidence from Japanese patients suggests that BP may develop in the presence of anti-LAD-1 IgG antibodies alone (21–24). Here, we present a case of BP which developed during treatment with nivolumab, in which anti-LAD-1 IgG antibodies alone were detectable. To our knowledge, this is the first case of checkpoint inhibitor-induced BP in a Caucasian associated with the production of anti-LAD-1 but absence of anti-BP180 NC16A IgG antibodies.

## Case Presentation

In 2009, a 69-year-old female was diagnosed with a superficial spreading melanoma (Clark level IV; 0.83 mm Breslow thickness; pT1b N3b M1a) of the right lower leg. The melanoma was surgically removed with a 1 cm safety margin. Six years later, in May 2015, the patient developed a subcutaneous in-transit metastasis on her right upper leg. The lesion was surgically excised and simultaneously a complete right inguinal lymph node dissection was performed. In October 2015, new inguinal, para-aortal, and iliac lymph node metastases, as well as new in-transit metastases, were radiologically detected. An immunotherapy with nivolumab (3 mg/kg) every 2 weeks was initiated based on the extent of the loco-regional disease. There was a rapid clinical response, with CT imaging evidencing a regression of the lymph node metastases by early February 2016 and only residual metastases remaining by April 2016.

**Figure 1:**
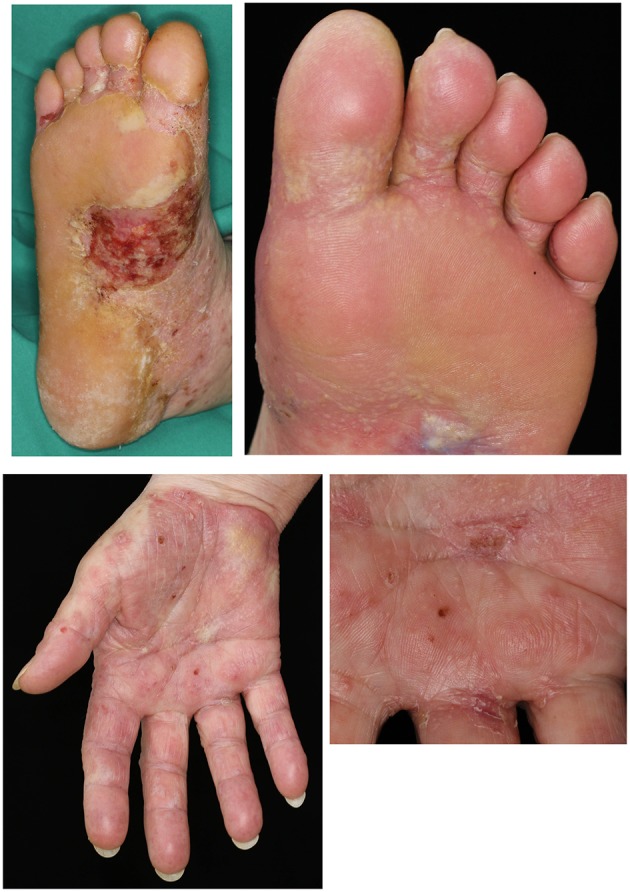
Initial clinical presentation of the cutaneous irAE with vesicles and pustules on the volar skin and the soles.

**Figure 2:**
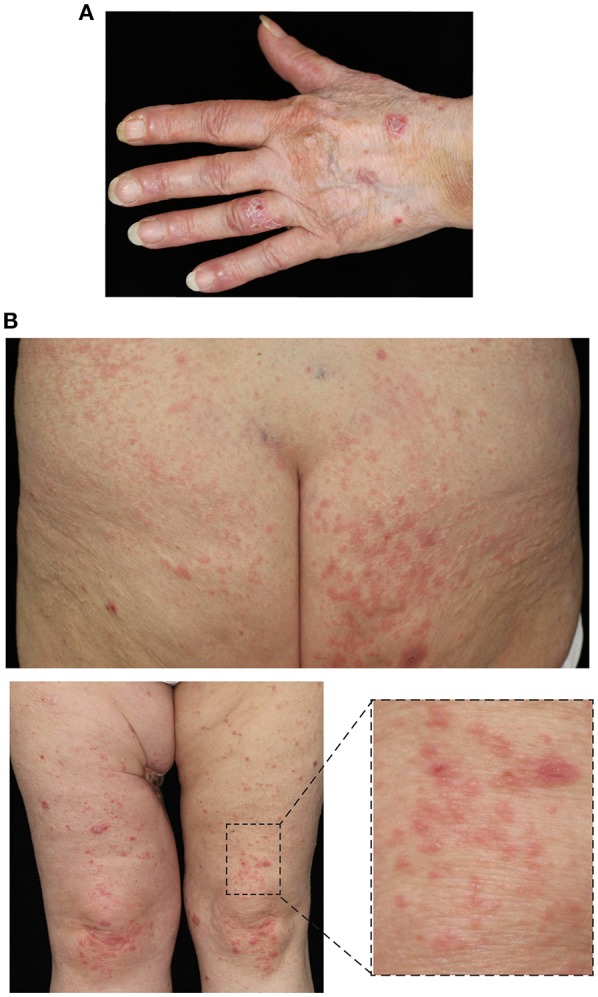
Progression of skin lesions with **(A)** blisters and erosions affecting the lower arms and dorsal hands and **(B)** lichenoid papules on the buttocks and thighs.

Prior to the diagnosis of melanoma, the patient had been otherwise fit with no significant co-morbidities. After 11 cycles of nivolumab, the patient developed thyroid peroxidase (TPO) autoantibodies, heralding the development of an immune-mediated thyroiditis which ultimately required substitution therapy with thyroxine. Concurrently, the patient developed small vesicles and pustules on the medial aspects of her palms and soles (Figure 1). Despite the application of topical corticosteroids (methylprednisolone cream) both the number and size of these lesions increased (Figure 2A). The patient also developed lichenoid papules and plaques on the legs and buttocks (Figure 2B). Given the clinical suspicion of an autoimmune blistering disorder, a diagnostic work-up including skin biopsies and serological testing was completed. Direct immunofluorescence (IF) microscopy of perilesional skin demonstrated linear, n-serrated disposition of IgG antibodies and C3 along the DEJ. There was no deposition of IgA autoantibodies.

**Figure 3:**
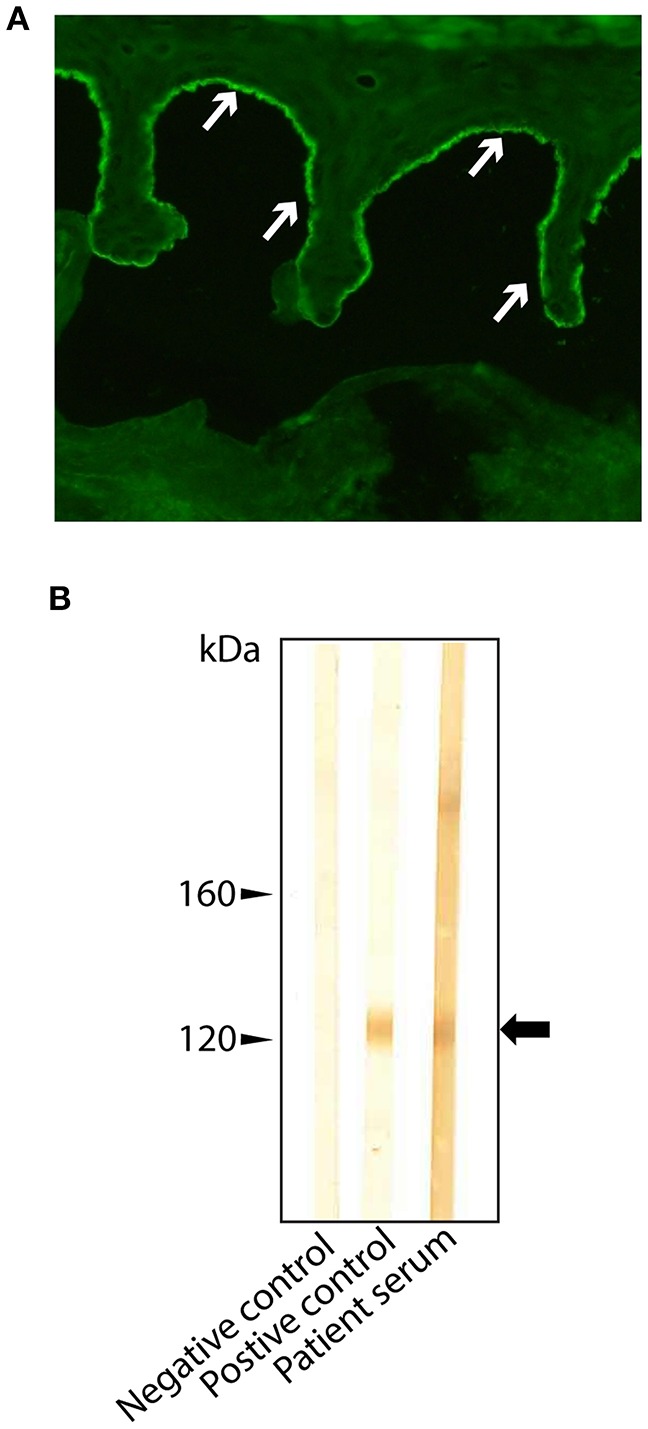
Routine laboratory diagnostic with **(A)** deposition of IgG (indicated by arrows) at the epidermal side of human split skin, and **(B)** detection of anti-LAD IgG autoantibodies by immunoblot on concentrated supernatant of cultured keratinocytes. The arrow indicates the signal identified as LAD-1.

In line with these findings, serum IgG antibodies were detected by indirect IF microscopy on both monkey esophagus and human salt-split skin (Figure 3A). IgA autoantibodies were not detectable. IgG was specifically deposited at the epidermal basement membrane zone on monkey esophagus and on the epidermal side of salt-split skin at a titer of 1:80. Interestingly, serum analysis for anti-BP180-NC16A and anti-BP230 IgG was negative using commercially available ELISA kits (EUROIMMUN, Lübeck, Germany). Therefore, the serum was subsequently examined for the presence of anti-LAD-1 IgG antibodies by immunoblot, using concentrated supernatant of cultured keratinocytes as the antigen, and revealed the presence of autoantibodies in the serum (Figure 3B).

Histopathologically, lesional skin exhibited a moderate, predominantly lymphohistiocytic dermal. Taken together, the clinical presentation and laboratory findings were consistent with the diagnosis of BP. The immune checkpoint therapy was discontinued after a total of 15 treatment cycles. Combined systemic (oral prednisolone, 50 mg per day; dapsone, 125 mg per day) and topical treatment (clobetasol propionate, twice daily on the entire integument) was initiated. The patient responded slowly to treatment with new blister formation ceasing after 4 weeks of treatment. The lichenoid papules also resolved slowly. The patient continued to suffer from severe pruritus, particularly on the feet, and developed vitiligo.

## Discussion

Cutaneous irAEs are the most common side effects of checkpoint inhibitors and can present with a range of skin manifestations; most frequently a non-specific dermatitis. However, our case suggests that in addition to increasing T-lymphocyte activation, the use of checkpoint inhibitors may also be associated with the development of highly specific, B cell-driven autoimmune responses. In fact, the growing number of case reports describing the emergence of anti-BP180 NC16A IgG autoantibodies in patients undergoing checkpoint therapy, the antibody most commonly responsible for the development of autoimmune blistering skin disease, supports this hypothesis. In our patient, however, the administration of nivolumab was associated with the induction of anti-LAD-1 IgG and the emergence of skin changes clinically and immunopathologically indistinguishable from BP. Anti-BP180 NC16A IgG was not detectable and neither was anti-BP230 IgG. With respect to the latter, we must draw our conclusions with the caveat that the ELISA kit used to determine anti-B230 IgG levels can only detect antibodies directed to the C-terminal end of the protein. Hence, we cannot exclude that antibodies against other epitopes of BP230 were generated in our patient and might have contributed to the eruption of BP.

Therefore, given that cutaneous irAEs may initially present with widespread pruritus and non-specific skin changes, patients should be closely monitored for the development of widespread urticarial, eczematous, and blistering skin lesions. Indeed, there should be a high index of suspicion that such patients have begun to develop, or will go on to develop, immunotherapy mediated-BP. Consequently, there should be a low threshold for obtaining a skin biopsy in patients with skin changes, particularly blistering lesions during checkpoint therapy, to determine whether linear deposition of IgG at the DEJ is present. Secondly, in addition to circulating anti-BP180 NC16A IgG antibodies, the presence of anti-LAD-1 IgG autoantibodies should also be determined.

Our case illustrates that merely determining circulating anti-BP180 NC16A IgG levels may not be sufficient to reliably diagnose checkpoint inhibitor mediated BP. Currently, whether some patients with checkpoint inhibitor-induced BP produce both anti-BP180 NC16A IgG and anti-LAD-1 IgG, as reported for a subset of spontaneous BP, is unknown. In this case, the determination of both anti-BP180 NC16A IgG and anti-LAD-1 IgG levels for diagnostic and disease monitoring purposes would be necessary.

Understanding the mechanisms through which checkpoint inhibitors induce BP may shed new light on the pathogenesis of spontaneous BP. For instance, our case supports the notion that anti-LAD-1 IgG alone is sufficient to induce skin blisters and erosions of significant severity. Intriguingly, most recently, a retrospective study in Japanese BP patients found that among those cases of BP, serologically negative for anti-BP180 NC16A IgG, 71% were positive for anti-LAD-1 IgG (17). This suggests that anti-LAD-1 IgG may be pathogenically more significant and more common than previously recognized. In line with previous studies, reporting that cases of BP missing anti-BP180 NC16A IgG autoantibodies tend to exhibit a lower degree of severity with respect to skin inflammation (25), in our case inflammation was on the milder than the average BP patient both on the clinical and the histopathological level.

As a consequence, anti-LAD-1 IgG should be assayed more frequently in the diagnostic workup of suspected cases of BP, particularly in the context of treatment with immune checkpoint inhibitors and when anti-BP180 NC16A IgG autoantibodies are not detectable.

Future studies should examine the extent to which the production of specific antibodies in BP (anti-BP180 NC16A IgG and/or anti-LAD-1 IgG) may actually affect treatment response, because there is first evidence that the emergence of cutaneous irAEs, particularly of vitiligo, may be associated with a better response to checkpoint inhibitor treatment (26). Furthermore, PD-1 activation may actually present a novel therapeutic strategy to suppress flares of BP, which could be investigated in future studies.

## Data Availability

The datasets generated for this study are available on request to the corresponding author.

## Ethics Statement

This study was carried out in accordance with the recommendations of the Ethics committee of the University of Lübeck with written informed consent from the subject. The subject gave written informed consent in accordance with the Declaration of Helsinki. The protocol was approved by the Ethics committee of the University of Lübeck.

## Author Contributions

CS, EL, VG, DZ, and PT analyzed and compiled the case. CS, EL, and PT wrote the manuscript. DZ edited the manuscript.

### Conflict of Interest Statement

The authors declare that the research was conducted in the absence of any commercial or financial relationships that could be construed as a potential conflict of interest.
